# Ag Nanocluster-Enhanced Scintillation Properties of Borophosphate Glasses Doped with CsPbBr_3_ Quantum Dots

**DOI:** 10.3390/ma15155187

**Published:** 2022-07-26

**Authors:** Ying Du, Lu Deng, Danping Chen

**Affiliations:** 1 Key Laboratory of Materials for High Power Laser, Shanghai Institute of Optics and Fine Mechanics, Chinese Academy of Sciences, Shanghai 201800, China; duying_ch@siom.ac.cn (Y.D.); denglu@siom.ac.cn (L.D.); 2 Center of Materials Science and Optoelectronics Engineering, University of Chinese Academy of Sciences, Beijing 100049, China

**Keywords:** glass-ceramics, Ag NCs, CsPbBr_3_ QDs, plasma effect, photoluminescence, radioluminescence

## Abstract

A novel and effective method to improve scintillation properties of glass-ceramics, such as intensity enhancement and decay-time shortening, is reported in this work. Compared with crystal scintillators, glass scintillators always have the problems of low efficiency and long decay; how to solve them has always been a scientific puzzle in the field of scintillation glass-ceramics. The plasma enhancement effect can be predicted to solve the above problems. Ag^+^ ions were diffused into glasses by ion exchange, and then Ag nanoparticles and CsPbBr_3_ quantum dots were formed by heat treatment. The structure of the CsPbBr_3_ perovskite consists of a series of shared corner PbBr_6_ octahedra with Cs ions occupying the cuboctahedral cavities. By using Ag and the plasma resonance effect, the photoluminescence intensity of CsPbBr_3_ quantum dot glasses was enhanced by 3 times, its radioluminescence intensity increased by 6.25 times, and its decay time was reduced by a factor of more than one. Moreover, the mechanism of photoluminescence and radioluminescence enhanced by Ag and plasma was discussed based on the experimental results and finite-difference time-domain method. We concluded that the increase in radioluminescence intensity was related to plasma enhancements and the energy exchange between Ag nanoclusters and CsPbBr_3_ quantum dots. Doping Ag is a valid means to improve the scintillation luminescence of CsPbBr_3_ quantum dot glasses, which can be applied in the field of scintillation.

## 1. Introduction

Scintillation materials can absorb ionizing radiation and emit photons in the visible or ultraviolet range. They are widely used in radiation detection applications, including medical diagnosis, safety inspection, industrial detection, basic scientific research, and radiation dosimetry [1,2,3,4,5]. A good scintillator should have a high light yield, fast decay time, high density, and low cost. Scintillation glasses have received considerable attention because they have low production costs and good optical uniformity. They can be prepared in large sizes and various shapes, such as being drawn into optical fibers [6,7,8,9]. The radioluminescence properties of Ce^3+^, Tb^3+^, Pr^3+^, Eu^2+^, and Yb^2+^ in various glasses have been extensively studied [10,11,12,13,14]. Except for Ce^3+^, the fluorescence lifetimes of the other ions are too long to be used as fast scintillation materials. Thus, Ce^3+^, which has the advantages of a nanosecond decay time, high light yield, and radiation resistance, has attracted the most attention and has been researched extensively. However, because of the charge migration quenching effect problem, low-density scintillation glasses doped with Ce^3+^ have high luminescence efficiency, while high-density glasses have low luminescence efficiency [15]. Hence, finding new scintillation luminescence centers to replace Ce^3+^ has become a critical issue. Cesium lead halide, CsPbX_3_ (X = Cl, Br, I), quantum dots (QDs) have attracted considerable attention because of their remarkable optoelectronic properties. In addition, some theoretical studies on CsPbX_3_ QDs by using first-principles calculations have also accomplished a great many achievements and laid the background for future experimental works. These theoretical studies have helped, directed, predicted and supported on important optics, luminescence properties, and structural features of CsPbX_3_ QDs [16,17]. However, their poor stability limits their practical applications [18,19]. Because a glass matrix can effectively prevent the degradation of metal halide perovskites, the stability of QDs embedded in glasses is significantly improved while still exhibiting good optical properties. Particularly, the luminescence of CsPbBr_3_ QDs in borophosphate glass at a temperature range of about 300 K to 500 K is stable in previous study [20]. To date, CsPbX_3_ perovskite QDs have been synthesized in low melting point phosphate oxyfluoride and borophosphate glasses [20,21], but the scintillation luminescence intensity of CsPbX_3_ QD-doped glasses is very low. This is because the size of the QDs is too small, and there are several defects on their surface. These defects act as recombination centers of electron-hole pairs generated by high-energy rays, thereby terminating energy transfer from electron-hole pairs to QDs. Therefore, improving the scintillation luminescence intensity of CsPbX_3_ QDs in glasses and reducing their fluorescence lifetimes are serious challenges for developing new scintillation glass-ceramics.

Luminescence properties of some materials can be enhanced by local surface plasmon effects caused by precious metal nanoparticles (NPs) [22,23]. Thus, it is imperative to introduce plasmonic nanostructures into glasses containing CsPbX_3_ QDs to improve their luminescence. Besides, macro-scale metal materials and their corresponding large-sized NPs will not produce fluorescent emission, but when the number of atoms or ions that make up the particles is reduced to a certain extent, nanoclusters (NCs) with discrete energy level structures and luminescence properties will be formed. These NCs do not have a complete lattice structure. Moreover, it is worth noting that the luminescence properties of NCs are closely related to their size distribution. As the number of atoms or ions that make up NCs increases (that is, the size of NCs increases), their excitation and emission spectra are redshifted, implying that NCs have size-dependent tunable luminescence properties [24,25,26]. One of the conditions for achieving resonance energy transfer is that the emission spectrum of the sensitizer and the excitation spectrum of the activator effectively overlap. Hence, how to flexibly and effectively regulate the emission band of metal NCs to achieve high-efficiency energy transfer for the activator is of great significance. Ag NPs can be introduced into glass in different ways. Compared with other methods, ion exchange has the advantages of low-cost, large-scale production, uniform particle distribution, and increased doping [27]. Xu et al. [28] had reported that the presence of these Ag-species resulted in enhanced photoluminescence total intensity by 5 times for the CsPbBr_3_ QD-doped glasses. Zhang et al. [29] had found that the 0.1 molar ratio Ag_2_O-doped CsPbBr_3_ quantum dot glasses sample had a photoluminescence intensity 2.37 times than the undoped sample. However, there is no report on the enhancement of scintillation luminescence of CsPbBr_3_ quantum dot glasses by Ag nanoclusters in the known literature. In this study, colorless and transparent borophosphate glasses were successfully prepared by melt-quenching. Appropriate ion exchange and heat-treatment processes were used to form Ag NPs and CsPbBr_3_ QDs with uniform particle sizes inside the glasses. Then, the photoluminescence and radioluminescence properties were measured and analyzed. Finally, the influence of the Ag NP content in glasses on the luminescence performance of CsPbBr_3_ QDs was simulated using the finite-difference time-domain (FDTD) method. Compared to other studies, the superiority, critical improvement, and novelty of the work lies in Ag plasma-enhanced scintillation luminescence of CsPbBr_3_ quantum dot glasses and reduced their fluorescence decay.

## 2. Experimental Procedures

### 2.1. Glass Composition and Preparation

**Figure 1:**
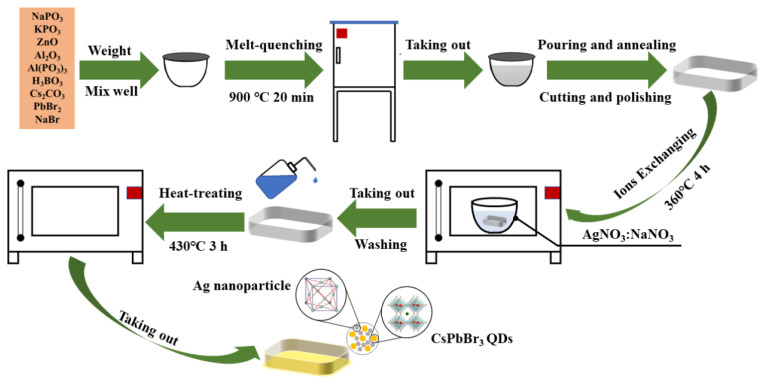
Schematic diagram of the preparation of PG glass and CsPbBr_3_ QD-doped glasses containing Ag NPs.

As shown in Figure 1, borophosphate glasses with a composition of 15P_2_O_5_-5Na_2_O-5K_2_O-10ZnO-10Al_2_O_3_-40B_2_O_3_-7Cs_2_O-3PbBr_2_-5NaBr (PG, the composition is expressed in mol.%) were prepared using the melt-quenching method. These glasses were proven to have excellent chemical stability and used for three-dimensional direct lithography of stable perovskite nanocrystals [21,30]. Starting materials were NaPO_3_, KPO_3_, ZnO, Al_2_O_3_, Al(PO_3_)_3_, H_3_BO_3_, Cs_2_CO_3_, PbBr_2_, and NaBr powders (analytical purity). They were thoroughly mixed in appropriate proportions and placed in covered corundum crucibles in 30 g batches. The melting conditions were 900 °C for 20 min, after which the glass melts were poured into a preheated stainless-steel plate. Each glass was annealed at 300 °C for 2 h in a muffle furnace to release the inner stress and then slowly cooled to room temperature. All obtained glass samples were cut and polished to a regular size of 10 × 10 × 2 mm^3^ for subsequent experiments.

Processed PG glasses were immersed in AgNO_3_:NaNO_3_ molten salts with different molar concentrations and heated at 360 °C for 4 h in a muffle furnace. The AgNO_3_ concentrations used were 0.25, 0.5, 1.0, 1.5, and 3.0 mol.%. After ion exchange, glass samples were washed with absolute ethanol and deionized water to remove residual nitrate from their surfaces. The obtained glass samples were denoted as IXP-0.25 Ag, IXP-0.5 Ag, IXP-1.0 Ag, IXP-1.5 Ag, and IXP-3.0 Ag. According to previous work [20], the PG glass transition temperature T_g_ was about 411 °C and the crystallization peak temperature T_p_ was about 555 °C. Generally, the heat-treatment temperature of glass-ceramic is selected between the T_g_ and the T_p_. Thus, the samples were then heated at 430 °C for 3 h in a muffle furnace to promote the formation of CsPbBr_3_ QDs and Ag NPs. Finally, the obtained glass-ceramics samples are denoted as HTP-0.25 Ag, HTP-0.5 Ag, HTP-1.0 Ag, HTP-1.5 Ag, and HTP-3.0 Ag. For comparison, a PG glass was directly heated at 430 °C for 3 h, and the obtained glass sample was denoted as HTP-0 Ag.

### 2.2. Characterization of Glass Samples

X-ray diffraction (XRD) patterns of glass samples were recorded on a Ultima IV X-ray diffractometer of Rigaku (Tokyo, Japan) using Cu Kα radiation, and the test range was 10–90° in steps of 10 °/min. X-ray photoelectron spectroscopy (XPS) spectra of glass samples were obtained using a K-Alpha X-ray photoelectron spectrometer of Thermal Fisher (Walthamm, MA, USA) and the bulk samples were polished on both sides. Before testing, the surface contaminants of glass samples were ultrasonically cleaned with ethanol, and these glasses were tested after drying. The microstructures of glass samples were observed using a JEOL 2100F transmission electron microscope (TEM) of JEOL (Tokyo, Japan) operated at an accelerating voltage of 200 kV and the glass samples were ground into powders, then placed in ethanol and sonicated for half an hour. Elemental distribution on glass sample surfaces was measured using a JXA-8230 electron probe microanalyzer (EPMA) of Japan and the bulk samples were polished on both sides and processed into flakes with a size of 10 × 10 × 1 mm^3^. The surface of glasses was cleaned and then carbonized. The Raman spectra of samples were tested using an InVia model Raman microscope of Thermo Fisher from (Walthamm, MA, USA), which excitation light source was a 785 nm argon-ion laser, the test range was 100–1000 cm^−1^, and the grating resolution was 1800 bars/mm. Absorption spectra were recorded using a Lambda 950 UV-Vis-NIR spectrophotometer of Perkin-Elmer from (Walthamm, MA, USA). Excitation and emission spectra and fluorescence lifetimes of glass samples were obtained using a FLS920 spectrophotometer of Edinburgh Instruments (Livingston, UK). Photoluminescence quantum yields (PLQYs), defined as the ratio of emitted photons to absorbed photons, were determined using a spectrofluorometer equipped with an 8 cm integrating sphere and a Xe lamp as the excitation source. X-ray-excited radioluminescence spectra were measured using an X-ray tube (Mo anode, 100 kV, 1 mA) with the SBP-300 fluorescence spectrometer of Zolix (Beijing, China).

## 3. Results and Discussion

**Figure 2:**
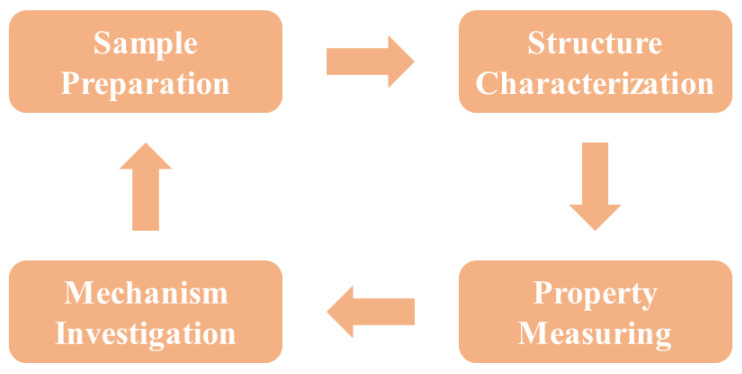
The program of research structure.

The purpose of the study is to improve the scintillation performance of CsPbBr_3_-QD-doped glasses. The research structure of the work including sample preparation, structure characterization, property measuring, and mechanism investigation is shown in Figure 2.

### 3.1. XRD Patterns of PG and CsPbBr_3_ QD-Doped Glasses Containing Ag NPs

**Figure 3:**
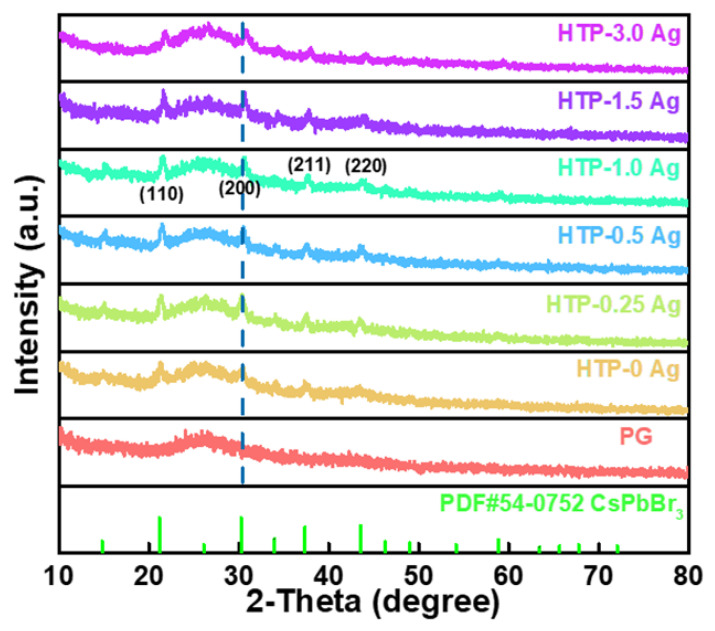
XRD patterns of PG and CsPbBr_3_ QD-doped glasses containing Ag NPs.

The XRD patterns of the PG glass and CsPbBr_3_ QD-doped glasses containing Ag NPs are shown in Figure 3. PG glass exhibits a typical amorphous structure, whereas distinct diffraction peaks gradually appear in CsPbBr_3_ QD-doped glasses containing Ag NPs. These main diffraction peaks were consistent with the (110), (200), (211), and (220) crystal planes of the cubic CsPbBr_3_ crystal (PDF #54-0752). Therefore, it can be inferred that CsPbBr_3_ crystals were successfully precipitated in the glasses after heat treatment. The XRD results also revealed that the addition of a small amount of Ag was favorable for the crystallization of CsPbBr_3_ QDs. However, as the concentration of AgNO_3_ was increased, the intensity of the diffraction peaks decreased, implying that the crystal quality of CsPbBr_3_ decreased. In addition, with the increase in AgNO_3_ concentration, the diffraction peaks of CsPbBr_3_ QD-doped glasses containing Ag NPs shifted to higher angles, indicating a decrease in interplanar spacing of crystal lattices. This may be caused by the partial replacement of Cs^+^ by Ag^+^ ions.

### 3.2. XPS Spectra of PG and CsPbBr_3_ QD-Doped Glasses Containing Ag NPs

**Figure 4:**
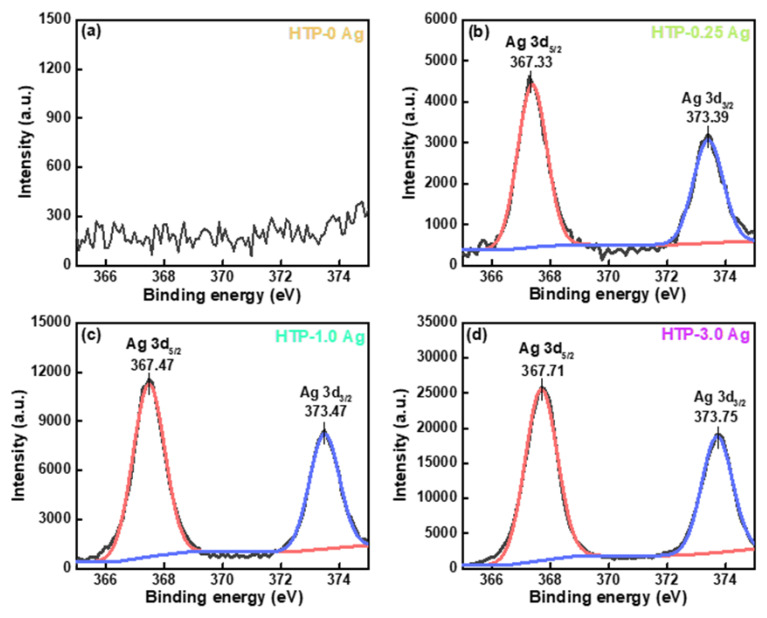
(**a**–**d**) Ag 3d XPS spectra of PG and CsPbBr_3_ QD-doped glasses containing Ag NPs.

The XPS spectra of the HTP-0 Ag, HTP-0.25 Ag, HTP-1.0 Ag, and HTP-3.0 Ag glasses are shown in Figure 4. The binding energy was calibrated by assuming C1s binding energy of 284.6 eV. Figure 4b–d show that CsPbBr_3_ QD-doped glasses containing Ag NPs have two prominent peaks near 367 and 373 eV, corresponding to the Ag 3d_5/2_ and Ag 3d_3/2_, respectively. According to the relevant literature, the approximately binding energy of 367–368 eV is attributed Ag 3d_5/2_ core shell of Ag^+^. The binding energy of 373–374 eV is attributed Ag 3d_3/2_ of metallic Ag [31,32]. Since the XPS binding energy of Ag NPs is slightly higher than that of Ag^+^ ions [33,34], both of them may be present. Compared with the *y*-axis of Figure 4b–d, the intensity of the two signal peaks increased with AgNO_3_ concentration, which indicates that the Ag NPs and Ag^+^ ion content in the glasses increased. Moreover, the binding energy of Ag 3d of CsPbBr_3_ QD-doped glass also increased with the AgNO_3_ concentration, implying that the proportion of Ag NPs increased.

### 3.3. TEM and EPMA Images of CsPbBr_3_ QD-Doped Glasses Containing Ag NPs

**Figure 5:**
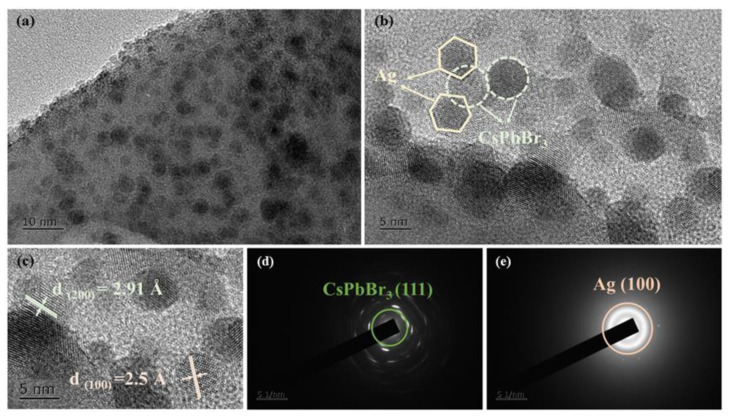
(**a**) TEM image, (**b**,**c**) HRTEM images, (**d**,**e**) SAED patterns.

The microstructure of CsPbBr_3_ QD-doped glasses containing Ag NPs was further characterized to confirm the successful precipitation of Ag NPs and CsPbBr_3_ QDs in the glasses. The TEM image of the HTP-1.0 glass (Figure 5a) demonstrates that numerous nanoparticles are evenly distributed in the glass matrix. Moreover, the high-resolution transmission electron microscopy (HRTEM, Figure 5b,c) confirms their single-crystal and highly crystalline nature with distinctly resolved lattice fringes. Meanwhile, a typical interplanar spacing of the (200) plane of an individual CsPbBr_3_ particle is 2.91 Å, while that of the (100) plane of Ag is 2.5 Å. In addition, as shown in Figure 5b, some CsPbBr_3_ QDs are surrounded by Ag NPs, which intuitively proves that the localized plasmonic effect generated by Ag NPs has an effect on CsPbBr_3_ QDs. Overall, the presence of both CsPbBr_3_ QDs and Ag NPs in glasses was revealed by TEM images. The selected area electron diffraction (SAED) patterns (Figure 5d,e) correspond to the diffraction images of the (111) plane of the CsPbBr_3_ crystal and the (100) plane of the Ag crystal.

**Figure 6:**
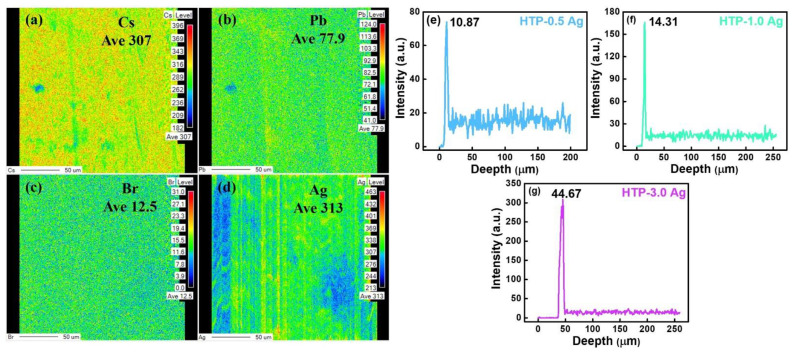
(**a**–**d**) the elemental maps of Cs, Pb, Br and Ag of HTP-1.0 Ag and (**e**–**g**) the depths of Ag^+^ ions in HTP-0.5 Ag, HTP-1.0 Ag and HTP-3.0 Ag glass samples.

Furthermore, the elemental maps of Cs, Pb, Br, and Ag on the surface of the HTP-1.0 Ag are shown in Figure 6a–d. Figure 6a–c shows that the distributions of Cs, Pb, and Br are relatively uniform in the same surface area, which indicates that these elements are uniformly dispersed in the glass. It further confirms that the CsPbBr_3_ QDs are uniformly distributed in the glass. Due to Br volatility, its content in the same area is far lower than that of Cs and Pb. However, as shown in Figure 6d, the content of Ag decreased in some areas, which indicates that after a long period of ion exchange, Ag^+^ ions diffused inside the glass. The depths of the Ag^+^ ions in HTP-0.5 Ag, HTP-1.0 Ag, and HTP-3.0 Ag were also measured (Figure 6e–g). The depth of Ag^+^ ions entering glasses was approximately ten to tens of micrometers from the glass surface, and the higher the concentration of AgNO_3_, the deeper the ions penetrated the glasses and the greater the content of Ag^+^ ions. The EPMA test results showed that the Ag element was on the surface and inside of glasses after ion exchange, and then, after heat treatment, Ag NCs would be formed on the surface and inside of glasses. Such overall crystallization glass-ceramics are beneficial for commercial applications.

### 3.4. Raman Spectra of CsPbBr_3_ QD-Doped Glasses Containing Ag NPs

**Figure 7:**
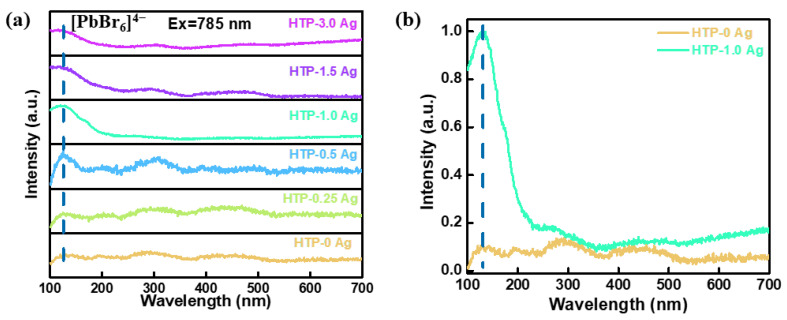
(**a**) Raman spectra of CsPbBr_3_ QD-doped glasses containing Ag NPs and (**b**) comparison spectra of Raman peak between HTP-0 Ag and HTP-1.0 Ag.

The Raman spectra of CsPbBr_3_ QD-doped glasses containing Ag NPs under a 785 nm laser excitation are shown in Figure 7a. The peak at 126 cm^−1^ gradually evolved for CsPbBr_3_ QD-doped glasses containing Ag NPs, corresponding to the second-order phonon mode of the [PbBr_6_]^4−^ octahedron [35,36]. This is another confirmation of the CsPbBr_3_ QD formation. Moreover, with an increase in the AgNO_3_ concentration, the vibration peak intensity of the [PbBr_6_]^4−^ octahedron tends to first increase and then decrease. When the concentration of AgNO_3_ is 1.0 mol.%, the peak at 126 cm^−1^ was the strongest. Compared with the HTP-0 Ag (without ion exchange), the peak intensity at 126 cm^−1^ of HTP-1.0 Ag increased by ca. 10 times. This may be due to the interaction between the surface plasmon resonance absorption effect of Ag NPs and CsPbBr_3_ QDs. Surface plasmons are ideal carriers for enhancing Raman scattering. Moreover, the energy of the light electric field is localized by Ag NPs within a very small area near the surface. Because the intensity of the Raman signal is proportional to the fourth power of the local electric field amplitude [37,38], the Raman signal peaks of CsPbBr_3_ crystals around Ag NPs are significantly enhanced. In the past, it was difficult to test the Raman peak of [PbBr_6_]^4−^ in CsPbBr_3_ QD-doped glasses. After adding Ag, the Raman peak intensity of [PbBr_6_]^4−^ could be significantly enhanced, which also reflected that Ag mainly entered around the quantum dots rather than around the glass phase, because the Raman peak intensity of the glass phase was not enhanced.

### 3.5. Photoluminescence Properties of CsPbBr_3_ QD-Doped Glasses Containing Ag NPs

**Figure 8:**
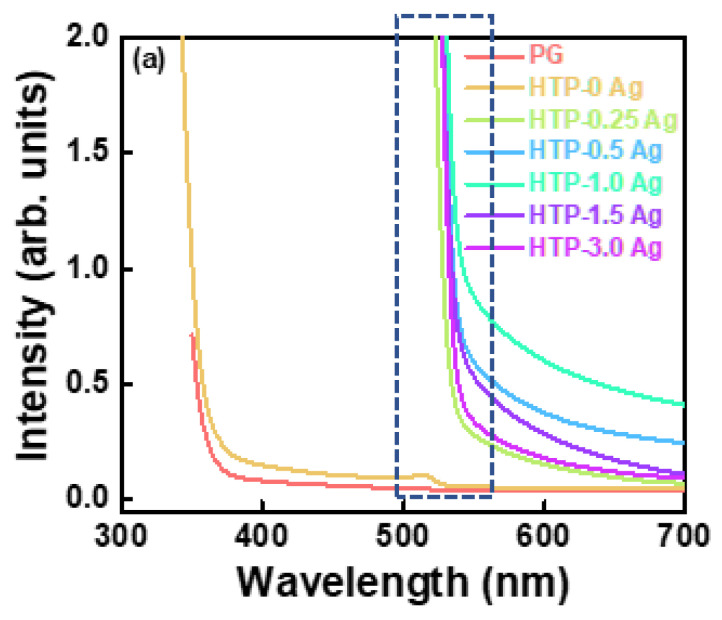

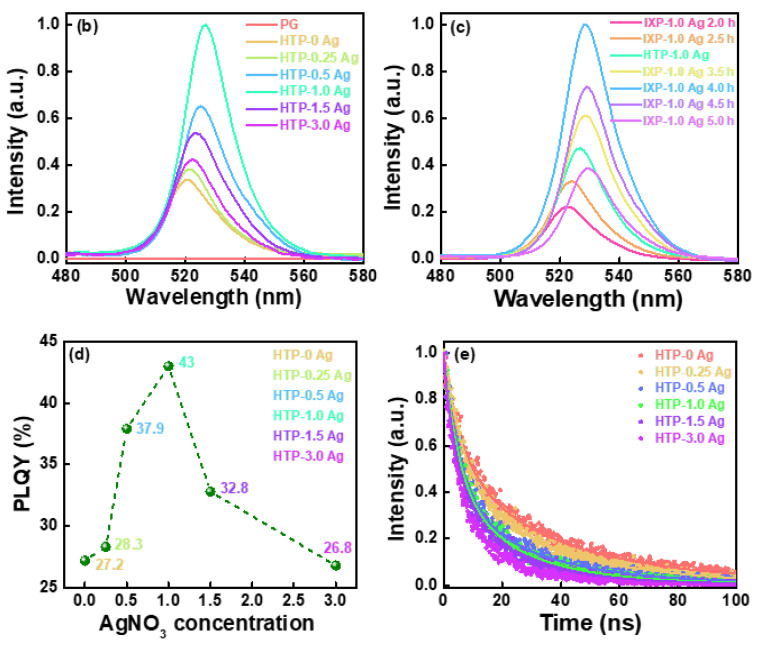
(**a**) Absorption, (**b**,**c**) emission spectra, (**d**) PLQYs and (**e**) fluorescence decay curves of CsPbBr_3_ QD-doped glasses containing Ag NPs.

The absorption spectra of PG and CsPbBr_3_ QD-doped glasses containing Ag NPs are shown in Figure 8a. The PG and HTP-0 Ag glasses are highly transparent in the visible range. The small absorption shoulder peaks of HTP-0 Ag near 515 nm were induced by CsPbBr_3_ QDs. In addition, CsPbBr_3_ QD-doped glasses containing Ag NPs exhibited no characteristic absorption peaks of Ag NPs. This may be because the absorption of QDs in CsPbBr_3_ QD-doped glass is too strong, resulting in the masking of the plasmonic absorption resonance peaks. Therefore, the absorption spectrum of HTB-1.0 Ag was measured (Figure S1). It shows the characteristic absorption peaks of Ag NPs in the 400–500 nm range. Compared with HTP-0 Ag, the absorption intensity of CsPbBr_3_ QD glasses containing Ag NPs significantly increased in the 500–550 nm range. This may be because the absorption ranges of Ag NPs and CsPbBr_3_ QDs coincide, and the surface plasmon resonance effect caused by Ag NPs significantly enhances the absorption of CsPbBr_3_ QDs surrounding the Ag NPs. In addition, according to the XRD results, the significant increase in the absorption intensity of CsPbBr_3_ QD glasses containing Ag NPs in the range of 500–550 nm may be also related to the substitution of Cs^+^ with Ag^+^ ions.

The fluorescence spectra of PG and CsPbBr_3_ QD-doped glasses containing Ag NPs under an excitation of 420 nm are shown in Figure 8b. All the CsPbBr_3_ QD-doped glasses containing Ag NPs exhibited a narrow-band emission of CsPbBr_3_ QD exciton recombination near 520 nm, which further proves the successful crystallization of CsPbBr_3_ particles. As the concentration of AgNO_3_ increased, the PL intensity of CsPbBr_3_ QD-doped glasses containing Ag NPs first increased and then decreased. When the concentration of AgNO_3_ was 1.0 mol.%, the PL intensity was the strongest. Compared to HTP-0 Ag, the photoluminescence intensity of HTP-1.0 Ag located at approximately 520 nm increased by approximately three times. This phenomenon can be explained by the strong coupling between the local surface plasmon resonance absorption of Ag NPs and the excitation light of 420 nm, which enhances the photoluminescence of CsPbBr_3_ QDs. It is well known that the intensity of plasmon resonance peaks depends on the concentration of the metal NPs in the glass matrix. With an increase in the Ag NP content in the glasses, the distance between the Ag nanostructures decreased and their interaction increased, which produced a local electric field and contributed to the photoluminescence enhancement. However, with a further increase in the Ag NP content, the PL intensity of the CsPbBr_3_ QDs decreased. This may be due to the following two reasons: (i) excessive Ag NPs inhibit the growth of CsPbBr_3_ QDs, and (ii) the plasmon absorption of excessive Ag NPs becomes a non-radiative relaxation pathway for photogenerated charge carriers. In addition, with the increase in AgNO_3_ concentration, the absorption cut-off wavelengths and the emission center wavelengths of CsPbBr_3_ QD glasses containing Ag NPs were first redshifted and then blueshifted. This is because adding a small amount of Ag can act as a nucleating agent to facilitate the crystallization of CsPbBr_3_ crystals. The increase in the size of the QDs causes a redshift in the absorption and emission spectra. However, as the concentration of AgNO_3_ was further increased, the absorption and emission spectra of CsPbBr_3_ QD glasses containing Ag NPs showed a blue shift, which implies that the grain size of CsPbBr_3_ decreased. There are two possible reasons for this. First, the mixed alkali effect caused by the addition of Ag^+^ inhibits the migration of Cs^+^ ions, making it difficult for CsPbBr_3_ QDs to grow. Second, a large number of Ag NPs enter the glasses, and too many crystal nucleating agents form too many crystallites, suppressing the growth of CsPbBr_3_ crystal grains. To further explore the photoluminescence properties of CsPbBr_3_ QD glasses containing Ag NPs, the fluorescence spectra of IXP-1.0 Ag under different heat-treatment times are shown in Figure 8c. The photoluminescence intensity of IXP-1.0 Ag 4 h increases approximately 2.2 times compared to that of the HTP-1.0 Ag. This indicates that the photoluminescence performance of CsPbBr_3_ QD-doped glasses containing Ag NPs can also be improved by precisely adjusting the heat-treatment process.

The PLQYs of the CsPbBr_3_ QD-doped glasses containing Ag NPs are shown in Figure 8d. As the concentration of AgNO_3_ increased, the PQLYs of the CsPbBr_3_ QD-doped glasses first increased and then decreased. HTP-1.0 Ag had a relatively high PLQY value. This can be attributed to changes in the size and quality of the CsPbBr_3_ crystals. Compared to HTP-0 Ag, the increase in the PLQY value of HTP-1.0 Ag is not positively related to the increase in its photoluminescence intensity. This is because CsPbBr_3_ QD-doped glasses containing Ag NPs have a stronger absorption of excitation light (Figure 8a).

The fluorescence decay curves of the CsPbBr_3_ QD-doped glasses containing Ag NPs are shown in Figure 8e. Owing to their non-single exponential features, the PL decay curves can be described by the following double exponential function:

*τ* = A_1_ exp(−*t*/*τ*_1_) + A_2_ exp(−*t*/*τ*_2_) (1)

The calculated average lifetimes of CsPbBr_3_ QD glasses containing Ag NPs are 24.90, 18.95, 14.95, 13.62, 12.25 and 8.99 ns, for HTP-0 Ag, HTP-0.25 Ag, HTP-0.5 Ag, HTP-1.0 Ag, HTP-1.5 Ag, and HTP-3.0 Ag glass samples, respectively. Compared with HTP-0 Ag, the fluorescence decay of CsPbBr_3_ QD-doped glasses containing Ag NPs was faster. As the Ag content increased, the fluorescence lifetime of the CsPbBr_3_ QD-doped glasses containing Ag NPs gradually decreased. This also confirms that the plasma effect increases the ability of the electrons to transition from a lower to a higher energy level. The rapid increase in the number of electrons at the upper energy level causes them to become unstable and the decay time becomes shorter. In other words, the plasma effect increases the PL intensity because it accelerates the number of cycles of electrons at the upper and lower energy levels.

**Figure 9:**
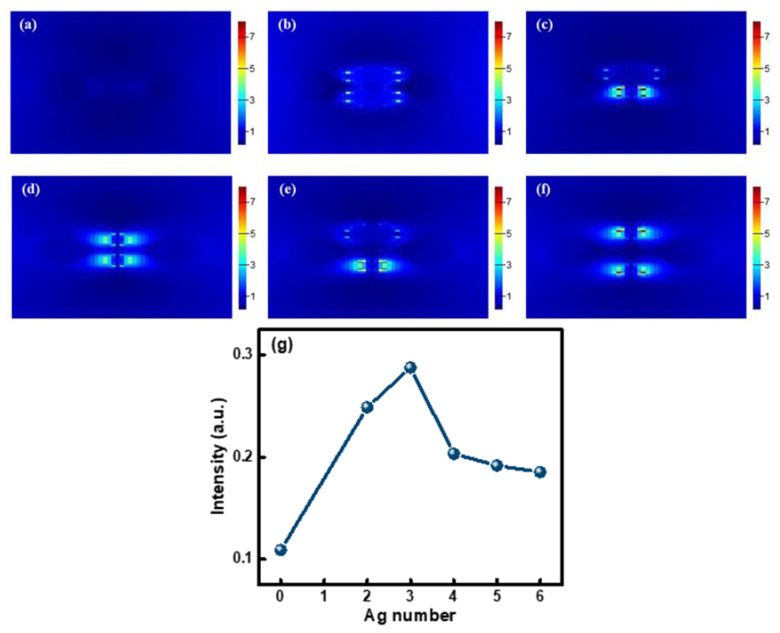
(**a**–**f**) Electric field intensity distribution of different numbers of Ag NPs in CsPbBr_3_ QD-doped glasses under excitation light calculated by using the FDTD method and (**g**) emitted light intensity of CsPbBr_3_ QD-doped glasses model containing different numbers of Ag NPs.

The influence of the Ag NP content on the photoluminescence properties of CsPbBr_3_ QDs in glasses was simulated using the FTDT method. A model was established based on the size and distribution of Ag and CsPbBr_3_ nanoparticles (Figure 5b). A rectangular parallelepiped was used to simulate a glass substrate, spheres were used to simulate Ag and CsPbBr_3_ nanoparticles, and a plane wave was used to simulate the excitation light. Perfectly matched layer boundary conditions were used to eliminate the influence of reflected light at the boundary to achieve the purpose of simulating an infinite space. An electric field monitor (E monitor), set above the model structure, was used to obtain the electric field distribution of the structure. An emitted light intensity monitor (T monitor) set above the model structure was used to obtain the emitted light intensity of the structure. The electric field intensity distributions of 0, 2, 3, 4, 5, and 6 Ag NPs around CsPbBr_3_ QDs in the established glass model under excitation light are shown in Figure 9a–f. As shown in Figure 9a, since there are no Ag NPs around the CsPbBr_3_ QDs in the glass model, only the electric field intensity distributions between CsPbBr_3_ QDs is exhibited, and its electric field intensity is very weak under excitation light. As there are more and more Ag NPs around CsPbBr_3_ QDs in the glass model, the electric field intensity between Ag NPs and CsPbBr_3_ QDs first increases and then decreases, as shown in Figure 9b–f. Moreover, the electric field intensity in Figure 9c is strongest. This phenomenon can be explained as the intensity of the plasmon resonance peak is related to the concentration of Ag NPs. In addition, the emitted light intensity of the CsPbBr_3_ QD-doped glass model containing different numbers of Ag NPs is shown in Figure 9g. With the increase in Ag NPs in the glass model, the emitted light intensity first increased and then decreased. Compared with the CsPbBr_3_ QD glass model without Ag NPs in Figure 9a, when there are three Ag NPs around CsPbBr_3_ QDs, the emitted light intensity increases by approximately three times. This result is consistent with the change in the electric field between Ag NPs in Figure 9a–f and the experimental results in Figure 8b.

### 3.6. Scintillation Properties of CsPbBr_3_ QD-Doped Glasses Containing Ag NPs

**Figure 10:**
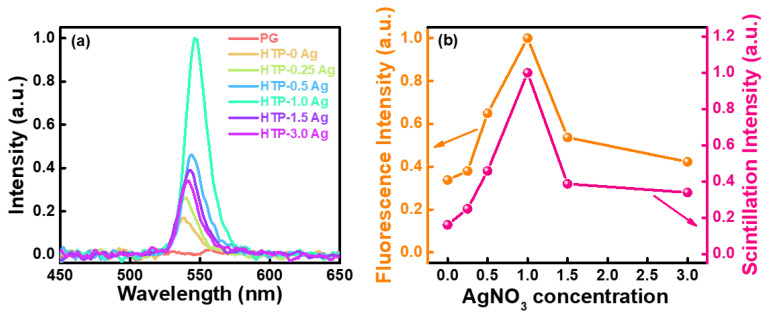
(**a**) X-ray excitation scintillation spectra of CsPbBr_3_ QD-doped glasses containing Ag NPs and (**b**) their radioluminescence and photoluminescence intensity comparison graph.

The X-ray excitation scintillation spectra of the CsPbBr_3_ QD-doped glasses containing Ag NPs are shown in Figure 10a. Under X-ray excitation, the PG glass had almost no radioluminescence emission. CsPbBr_3_ QD-doped glasses containing Ag NPs had a narrow-band emission near 540 nm, and with an increase in the content of Ag NPs, their scintillation intensity first increased and then decreased. HTP-1.0 Ag had the strongest radioluminescence. These results are consistent with previous photoluminescence results. The radioluminescence and photoluminescence intensity comparison graph of CsPbBr_3_ QD-doped glasses containing Ag NPs is shown in Figure 10b. Compared with HTP-0 Ag, the photoluminescence intensity of HTP-1.0 Ag glass increases by approximately 3 times, while its radioluminescence intensity increases by approximately 6.25 times. This may be because Ag NCs are composed of Ag^+^ ions, and Ag NPs emit light under ultraviolet short-wavelength excitation. This can enhance the radioluminescence of CsPbBr_3_ QDs in glass through energy transfer. To support this conjecture, the excitation spectra of HTP-0 Ag and CsPbBr_3_ QD-doped glasses containing Ag NPs were measured and analyzed (Figure S2). Unfortunately, the energy transfer between Ag NCs and CsPbBr_3_ QDs was not observed in their excitation spectra and the corresponding emission spectra in Figure 8b. This is because the absorption of CsPbBr_3_ QDs is much stronger than that of Ag NCs. Therefore, the excitation and emission spectra of the HTB-1.0 Ag glass were measured (Figure S3a,b). Under ultraviolet short-wavelength excitation, HTB-1.0 Ag exhibits broadband luminescence in the ultraviolet-visible band, which is attributed to the luminescence of Ag NCs. The Ag NCs in the glass is formed by agglomeration and assembly of Ag NPs and Ag^+^ ions. At present, the geometry and energy level structure of Ag NCs that can produce radiation transitions in glass matrices of different components have not been fully clarified. It is generally believed that the clusters with radioluminescence properties may be Ag_2_^+^, Ag_3_^2+^, Ag_4_^2+^, etc. [39,40,41]. The PL and radioluminescence properties of CsPbBr_3_ QD-doped glasses containing Ag NPs are summarized in Table 1. Obviously, the plasma effect caused by the injection of Ag NPs can enhance the scintillation luminescence intensity of CsPbBr_3_ QD-doped glasses and reduce their fluorescence lifetimes.

## 4. Conclusions

Scintillation glasses and glass-ceramics generally have the problems of low light yield and long decay time, which is the dilemma and bottleneck in scintillation glasses research at present. To solve these problems, Ag^+^ ions were first diffused into glasses by ion exchange, and then Ag NCs and CsPbBr_3_ QDs were formed in transparent glass-ceramics by heat treatment. Compared with CsPbBr_3_ QD glass, the photoluminescence intensity of Ag^+^-doped CsPbBr_3_ QD glass was enhanced by 3 times, its radioluminescence intensity increased by 6.25 times, and its decay time was reduced by a factor of more than one via the above process. Moreover, the mechanism of photoluminescence and radioluminescence enhanced by Ag and plasma was discussed based on the experimental results and finite-difference time-domain method. Ag NCs not only greatly enhanced the light yield of CsPbBr_3_ QD-doped glass but also effectively shortened their fluorescence decay time, thereby considerably improving the scintillation property of CsPbBr_3_ QD-doped glass. This work provides a new path for the development of new scintillation glass-ceramic materials. With further optimization of glass composition and the concomitant increase in density, CsPbBr_3_ QD-doped glasses containing Ag NCs can be used in fast scintillation devices such as colliders for scientific experimental research in high-energy physics and nuclear physics.

## Figures and Tables

**Table 1:** Scintillation performances of CsPbBr_3_ QD-doped glasses containing Ag NPs.

Glass Sample	Photoluminescence Center Wavelength (nm)	Photoluminescence Peak Intensity (a.u.)	Radioluminescence Center Wavelength (nm)	Radioluminescence Peak Intensity (a.u.)	Fluorescence Lifetime (ns)	PLQY (%)	Density (g/cm^3^)
HTP-0 Ag	520	0.34	538	0.16	24.90	27.2	2.75
HTP-0.25 Ag	521	0.38	540	0.26	18.95	28.3	2.82
HTP-0.5 Ag	525	0.65	543	0.45	14.95	37.9	2.91
HTP-1.0 Ag	526	1	547	1	13.62	43.0	3.03
HTP-1.5 Ag	523	0.54	542	0.39	12.25	32.8	3.12
HTP-3.0 Ag	522	0.42	541	0.34	8.99	26.8	3.24

## Data Availability

The study did not report any data.

## Supplementary Materials

The following supporting information can be downloaded at: https://www.mdpi.com/article/10.3390/ma15155187/s1, Figure S1: Absorption spectrum of HTB-1.0 Ag. Figure S2: Excitation spectra of PG and CsPbBr3 QD-doped glasses containing Ag NPs. Figure S3: (a) Excitation and (b) emission spectra of HTB-1.0 Ag
