# Supplementary material for: Ag Nanocluster-Enhanced Scintillation Properties of Borophosphate Glasses Doped with CsPbBr3 Quantum Dots
Source: Materials (Basel). 2022 Jul 26;15(15):5187. doi: 10.3390/ma15155187 (PMC9330410; doi:10.3390/ma15155187)
Supplement: Supplementary file 1 [file materials-15-05187-s001.zip › materials-1811886-supplementary.pdf]

materials

MDPI

Ag nanocluster-enhanced scintillation properties of borophosphate glasses doped with $CsPbBr_3$ quantum dots

Ying Du[1,2], Lu Deng[1], Danping Chen[1*]

[1]Key Laboratory of Materials for High Power Laser, Shanghai Institute of Optics and Fine Mechanics, Chinese Academy of Sciences, Shanghai 201800, P. R. China

[2] Center of Materials Science and Optoelectronics Engineering, University of Chinese Academy of Sciences, Beijing 100049, P. R. China

*Email: d-chen@mail.siom.ac.cn

*Postal address: Shanghai Institute of Optics and Fine Mechanics, Shanghai, China

For comparison, borophosphate glasses with a composition of $15P_2O_5$-$5Na_2O$-$5K_2O$-10ZnO-$10Al_2O_3$-$40B_2O_3$-$7Cs_2O$-5NaBr (BPAZNK, the composition is expressed in mol.%) were prepared using the melt-quenching method. The processed BPAZNK glasses were subjected to the same ion exchange as the PG glasses. The obtained glass samples were denoted as IXB-0.25 Ag, IXB-0.5 Ag, IXB-1.0 Ag, IXB-1.5 Ag, and IXB-3.0 Ag. The samples were then heated at 430 °C for 3 h in a resistance furnace. Finally, the obtained glass samples are denoted as HTB-0.25 Ag, HTB-0.5 Ag, HTB-1.0 Ag, HTB-1.5 Ag, and HTB-3.0 Ag.

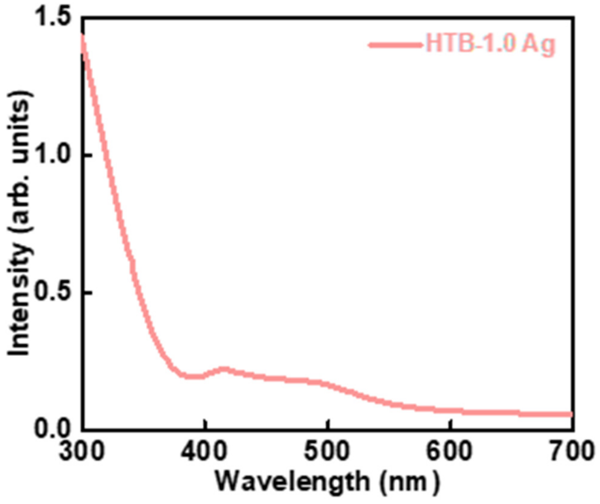

Figure S1. Absorption spectrum of HTB-1.0 Ag.

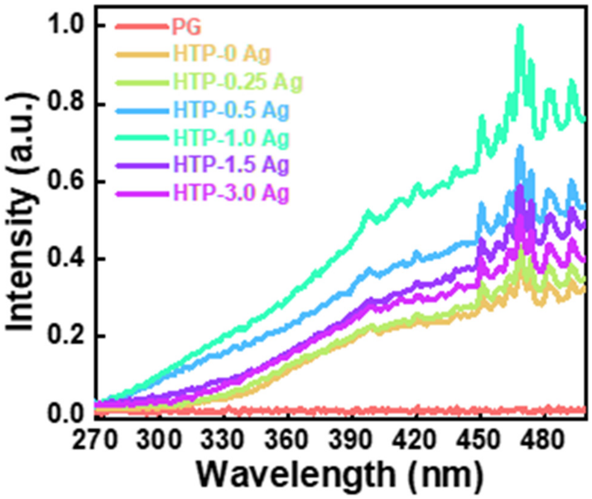

Figure S2. Excitation spectra of PG and $CsPbBr_3$ QD-doped glasses containing Ag NPs.

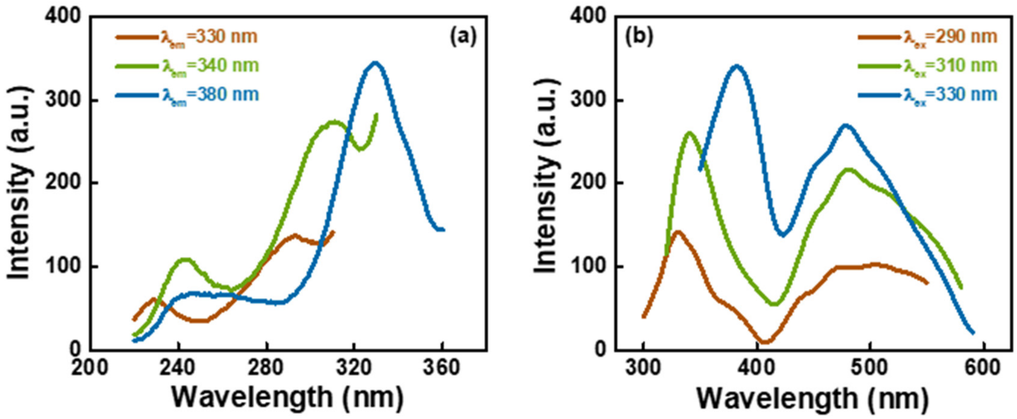

Figure S3. (a) Excitation and (b) emission spectra of HTB-1.0 Ag.
